# Finger enchondroma treated with bone substituents – a case presentation

**Published:** 2014-06-25

**Authors:** L Raducu, A Anghel, S Vermesan, RD Sinescu

**Affiliations:** * Department of Plastic Surgery, "Agrippa Ionescu" Military Emergency Hospital, Bucharest, Romania; ** Department of Plastic Surgery and Reconstructive Microsurgery, "Elias" Emergency University Hospital, "Carol Davila" University of Medicine and Pharmacy, Bucharest, Romania; *** Department of Orthopedic Surgery, "Agrippa Ionescu" Military Emergency Hospital, Bucharest, Romania

**Keywords:** enchondroma, bone tumor surgery, bone grafting, bone substituents

## Abstract

Abstract

About 90% of the hand bone tumors are enchondromas. Treatment of choice is complete curettage and bone grafting, usually with bone autografts. We present a case of finger enchondroma in a 27-year-old female patient who was treated with curettage and synthetic bone grafting. Clinical, surgical and pathological findings in this case are presented along with a brief discussion of literature.

## Introduction

Enchondromas are benign cartilaginous lesions predominantly seen in the skeleton of the hand (approximately 35% of all enchondromas develop in the hand) and are the most frequent (as many as 90%) osseous tumors of the hand [**1,2**].

 Enchondromas may present as solitary, monostotic lesions with a peak incidence in the fourth decade and with a predilection for the short tubular bones (proximal phalanx, metacarpal / metatarsal, middle phalanx) of the hands and feet, distal femur, and proximal humerus, or as multiple / polyostotic lesions in Ollier's disease (enchondromatosis), a non-heritable condition, and in Maffucci's syndrome, a combination of multiple enchondromas and hemangiomas. Malignant degeneration of monostotic enchondroma to chondrosarcoma, although rare, has been well described [**1**].

 Given the fact that individuals with enchondromas usually have few symptoms and signs (typically a localized painless swelling), or sometimes none at all, an enchondroma may be diagnosed during a routine physical examination, as an incidental finding on plain radiographs, or in the event of a pathologic fracture commonly caused by minor trauma and favored by the presence of the tumor.

 Radiographs typically demonstrate a well-defined lytic lesion, central or eccentric, expansive or not, habitually containing calcified chondroid matrix and non-invading into the surrounding tissue. Additional diagnostic imaging for enchondroma may include radionuclide bone scan, magnetic resonance imaging (MRI) and computed tomography scan (CT), but in the overwhelming majority of cases, plain radiographs are sufficient for the diagnosis [**1**].

 While the attitude in small asymptomatic lesions may be conservative, consisting of a simple observation, curettage has recently become the mainstay of surgical treatment for the large or symptomatic enchondromas. If the enchondroma is diagnosed as a pathologic fracture, the lesion may be treated immediately or after the fracture has healed. Although several authors recently reported excellent results with simple bone curettage, the bone defects resulting from curettage of enchondroma are usually filled with autologous bone grafts (e.g., iliac crest, distal radius), fresh frozen or freeze-dried irradiated allografts, or bone substitutes (calcium phosphate, calcium sulphate, tricalcium phosphate, hydroxyapatite). These newer materials eliminate the morbidity of the bone graft harvest site and lower the disease’s transmission risk of the allograft [**3**].

 Patient, methods and result

**Fig. 1:**
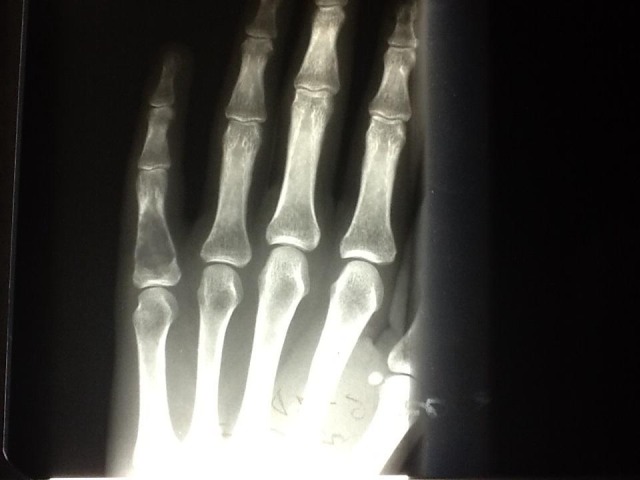
Frontal radiography of little finger

 We present a case of a young patient with a finger enchondroma investigated, diagnosed and treated with bone substituents. A 27-year-old woman was admitted in our Department with one-week history of pain and moderate swelling on the fifth right finger’s proximal phalanx. The pain had a sudden onset after the patient opened her car's door. There was no significant history of smoking or alcohol consumption or trauma. Clinical examination revealed a painful palpable mass on the phalanx, crepitation and limited passive/active PIP flexion. Frontal radiography of little finger revealed a lytic expansive lesion on the proximal phalanx associated with thinning of the bone cortex and a pathological fracture at the same level (**Fig. 1**).

**Fig. 2:**
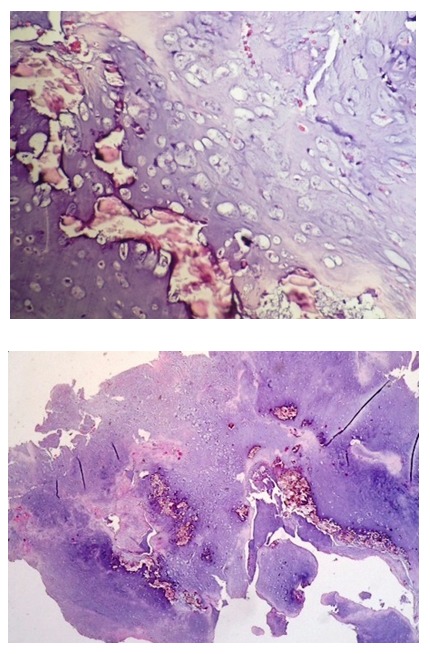
The pathology exam confirmed the diagnosis of enchondroma

Because of the small incidence of malignant transformation associated with pathologic fractures [**4**], we decided to curettage the tumor and wait for the pathology result. We used a dorsal approach and an appropriate-sized cortical window to expose the tumor and we removed it by curettage. The pathology exam showed mature lobules of hyaline cartilage, foci of calcification and enchondreal ossification, which confirmed the diagnosis of enchondroma (**Fig. 2**). 

**Fig. 3:**
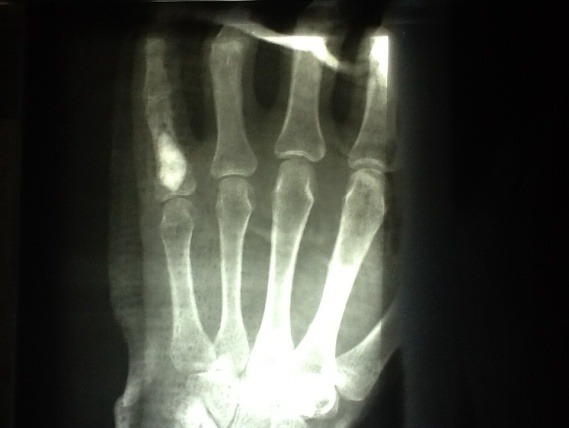
Injection with high compressive strength

 For the filling of the resulted bone defect, we decided to use a bone graft substitute because of the lack of complications and donor-site morbidity, ease of use and rapid local improvement. We used CaSO4 minimally invasive injectable graft (MIIGX3), with faster resorption and bone repopulation. The substance consolidated in situ several minutes after the injection with high compressive strength (**Fig. 3**). 

**Fig. 4:**
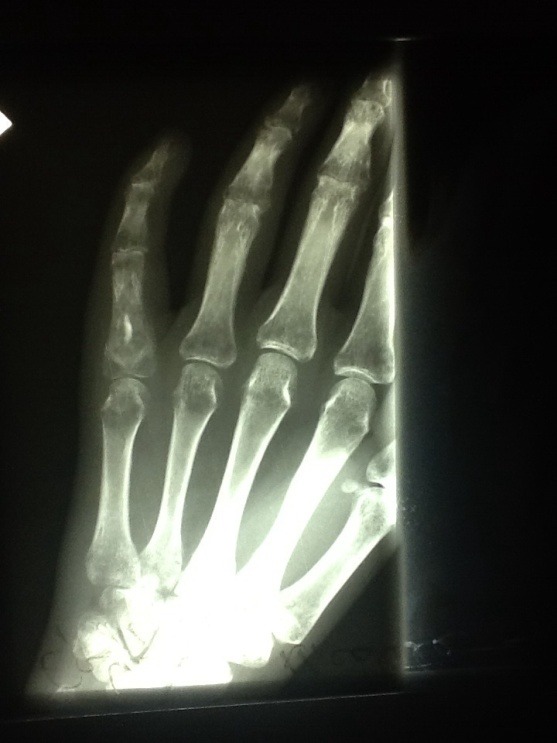
Radiography at 4 weeks postoperatively

The patient maintained finger spica split for 4 weeks; afterwards the range of motion therapy was initiated. Radiography at 4 weeks postoperatively revealed a well-mineralized bone (**Fig. 4**). 

 At 8 weeks from surgery, the patient showed normal flexion-extension movements in the PIP joint.

## Discussion

Bone grafting is an essential procedure in musculoskeletal tumor surgery, more than 2.2 million bone grafts being performed annually for the repair of bone defects and approximately 10% of all skeletal reconstructive surgery requiring bone grafting [**5**]. The large defects created after bone tumor curettage may require bone grafts (autogeneic, allogeneic or synthetic) as part of the treatment. 

 Autologous bone graft from the iliac crest, cortical or cancellous, is the most current choice, but it is associated with a 8-39% complication rate (infection, hematoma, urethral injury, pelvic instability, cosmetic disadvantages, chronic pain) [**3**] and with a risk of donor-field contamination with tumor cells. 

 The use of bone allografts, available in various shapes and sizes, decreases the operating time and does not associate any donor-site morbidity or complications. Nevertheless, since the allografts are harvested from deceased donors, a small risk of transmission of infectious agents still exists, despite the strict measures of tissue processing and sterilization applied. 

 All the above-mentioned limitations of bone auto- and allografts stimulated the development of alternatives (synthetic) bone substitutes, currently numerous products such as hydroxyapatite, bicalcium or tricalcium phosphate, calcium sulphate, BMP2, BMP7 being available). Each of these substances has its characteristics, indications and specific uses, as demonstrated by recent clinical studies. 

 Dreesmann made the first reported use of CaSO4 in 1892, filling cavities in tuberculosis patients [**6**]. Nowadays, CaSO4 is available in the form of paste, granules or blocks. It functions as a resorbable osteoconductive scaffold, fully dissolving in 6-12 weeks, that provides the structural framework necessary for angiogenesis and osteogenesis. It is mainly used to fill gaps resulted from tumor resection and periarticular fractures, but it does not offer sufficient structural support to be utilized in minimal weight baring bones [**3**].

## Conclusions

We report a case of a finger bone tumor believed to be an enchondroma at presentation. In our case, the presence of a pathological bone fracture and the width of the tumor warranted the need of bioptic curettage, since the tumor could have been either an enchondroma or a low-grade central chondrosarcoma. The histopathological exam confirmed the enchondroma diagnosis. 

 Our patient was a young woman who did not accept the donor site comorbidities of a bone autograft, so we offered her the possibility of using a bone substitute instead. 

 Curettage and CaSO4 injected graft (MIIGX3) proved to be a safe, promising treatment modality for hand enchondroma. It led to an excellent functional and radiological result, as it reduced the operating and recovery time and had no donor site morbidity. Further studies are necessary to demonstrate its worth as a standard choice of treatment for hand bone defects. 
